# The impact of age on prostate cancer progression and quality of life in active surveillance patients

**DOI:** 10.1002/bco2.52

**Published:** 2020-11-29

**Authors:** Gregory S. Merrick, Gabe Rohmann, Robert Galbreath, Whitney Scholl, Ryan Fiano, Abbey Bennett, Wayne M. Butler, Edward Adamovich

**Affiliations:** ^1^ Schiffler Cancer Center Urologic Research Institute Wheeling WV USA; ^2^ Department of Urology Wheeling Hospital Wheeling WV USA; ^3^ Ohio University Eastern St Clairsville OH USA; ^4^ Department of Pathology Wheeling Hospital Wheeling WV USA

**Keywords:** active surveillance, patient age, prostate cancer, quality of life, transperineal template‐guided mapping biopsy

## Abstract

**Objectives:**

To evaluate the impact of age on overall survival (OS), freedom from distant metastasis (FDM), rates of therapeutic intervention (TI), and quality of life (QOL) in active surveillance (AS) prostate cancer patients.

**Materials and methods:**

Three hundred and five consecutive, prospectively evaluated AS patients who underwent a staging transperineal template‐guided mapping biopsy (TTMB) prior to enrollment on AS were evaluated and stratified by age. Evaluated outcomes included OS, FDM, TI, and QOL to include urinary, bowel, sexual function, and depression. Post void residual (PVR) urine measurements were also followed. Repeat biopsy was based on PSA kinetics, abnormal digital rectal examination or patient preference.

**Results:**

Of the 305 patients, 290 (95.1%) were Gleason 3 + 3 and 15 patients (4.9%) were Gleason 3 + 4. The median follow‐up was 5.5 years (range 1‐14 years). At 10 years, TI was 0%, 1.0%, and 11.4% for patients ≤59, 60‐69, and ≥70 years of age (*P* < .001). No patient has developed distant metastasis. The median time to TI was 4.71 years. No statistical differences in urinary function, bowel function, or depression were noted. Potency preservation was dependent on patient age.

**Conclusion:**

Within the confines of the follow‐up of our series, younger patients were less likely to proceed to therapeutic intervention. In addition, patient age did not adversely impact QOL outcomes.

## 1 INTRODUCTION

Active surveillance (AS) represents a paradigm change in the management of low‐risk prostate cancer. In appropriately selected patients, AS has been demonstrated to reduce the over‐treatment of indolent prostate cancers with improvement in quality of life (QOL) with the caveat that the diagnosis of lethal malignancies remain feasible while the cancer remains curable.^1^, ^2^ Multiple AS studies have documented prostate cancer specific survival rates of 94‐99.9% at 15 years with non‐prostate cancer deaths being 9‐24 times more likely.^3^, ^4^

AS protocols are hampered by a lack of consensus regarding standardized criteria for inclusion, follow‐up, and the initiation of therapeutic intervention.^3^, ^4^, ^5^ In particular, definitive local therapy is often recommended to healthy younger men with low‐risk features due to a long life expectancy. Although younger patients may potentially be at greater risk for prostate cancer death without local therapy, they also are at greater risk for adverse QOL outcomes to include urinary, bowel, and sexual function when compared to older patients.^6^ Leapman and colleagues evaluated 1,433 AS patients with the conclusion that at 5 years patients ≤60 years of age were less likely to require therapeutic intervention.^7^ Mahal et al in an evaluation of the Surveillance, Epidemiology and End Results (SEER) database reported that young men are increasingly managed with active surveillance with AS rates increasing from 22% in 2010 to 58% in 2015.^8^ The 5 year prostate cancer specific mortality rates were <0.30% across all age cohorts regardless of upfront treatment management strategies.^8^

Transperineal biopsy techniques systematically map the prostate gland and provide more accurate information regarding prostate cancer grade, volume, spatial distribution of cancer, and decrease the infectious morbidity of transrectal ultrasound (TRUS) biopsy. The possibility of missed high‐grade prostate cancer at initial biopsy poses the greatest risk to AS patients in terms of lost opportunity for cure.^9^ Approximately 1/3 of AS patients proceed to therapeutic intervention within the first few years of diagnosis, most likely a result of sampling error due to TRUS biopsy. TTMB has been demonstrated to accurately identify high‐grade cancers when compared to whole‐mount radical prostatectomy (RP) pathology.^10^ The majority of clinically significant prostate cancers that are missed at TRUS biopsy are located in the anterior prostate.^11^, ^12^ Previously, in a TTMB staging series we reported that Gleason score is upgraded in 39% of patients with upgrading most common in the anterior prostate and apex.^13^, ^14^

In our series, all patients undergo a transperineal template‐guided mapping biopsy (TTMB) prior to AS enrollment.^4^ In the current study, we evaluated the impact of patient age on AS outcomes to include overall survival (OS), freedom from distant metastases (FDM), therapeutic intervention (TI), post void residual urine (PVR) and urinary, bowel, sexual, and depressive quality of life parameters.

## 2 MATERIALS AND METHODS

From April 2005 through June 2017, 305 consecutive, prospectively evaluated patients underwent TTMB for TRUS or transperineal diagnosed Gleason score 6 (3+3) prostate cancer, a persistently elevated PSA or the presence of atypical small cell acinar proliferation (ASAP) and were deemed eligible for AS. Eligibility was defined as clinical stage T1c, Gleason score 6 (3 + 3) and a PSA density <0.22 ng/mL per cm^3^ or Gleason score 7 (3+4) with ≤ 3 positive biopsies, a PSA < 10 ng/mL and a patient age ≥ 70 years.^3^ Follow‐up consisted of serial PSA’s every 4 months, yearly digital rectal examinations (DRE) and serial QOL evaluations. Therapeutic intervention and/or repeat TTMB biopsy was recommended for a PSA doubling time <3 years, a change in DRE and/or patient preference.

Previously, the TTMB technique has been described in great detail.^13^, ^14^ All biopsies were performed by a single operator (GSM). Two days prior to TTMB tamulosin (0.8 mg daily) was initiated and continued for 2 weeks. TTMB was performed in the operating room with the patient in dorsal lithotomy position under general anesthesia. All patients received peri‐operative antibiotics. The prostate gland was scanned from the proximal seminal vesicles/base of the prostate gland to the apex. A volumetric ultrasonographic evaluation was obtained to determine prostate size. In addition, the prostate gland and transition zone (TZ) volumes were estimated as an ellipsoid with the formula: length × width × height × π/6.

Transperineal biopsies were obtained through template apertures corresponding to the 24 regional biopsy locations.^13^, ^14^ For each of the 24 regions, as many as four biopsy cores were taken, depending on prostate size. Eighteen gauge, 25 cm long Max‐Core biopsy needles (C.R. Bard Inc., Covington, GA, USA) were used. For each biopsy core, the template coordinate, and the offset from the base were recorded. Biopsies were taken to sample the entire gland including the posterior (sites 3, 4, 12, 13, 21, 22), posterior lateral (sites 2, 5, 11, 14, 20, 23), anterior lateral (sites 1, 6, 9, 10, 15, 16), anterior apex (sites 19, 24), and transition zone (TZ) (sites 7, 8, 17, 18). All pathologic assessment was performed by a pathologist with significant expertise in prostate pathology (EA).

Clinical parameters included overall survival (OS), therapeutic intervention (TI), freedom from distant metastases (FDM) and prostate cancer specific mortality (PCSM). Cause of death was verified for each dead patient. PSA kinetics were evaluated as per Vickers et al^15^ and the Sengupta definition.^16^ At initial consultation and at every follow‐up visit, patients completed the International Prostate Symptom Score (IPSS),^17^ the Rectal Function Assessment Score (R‐FAS),^18^ the International Index of Erectile Function‐6 (IIEF‐6),^19^ the Center for Epidemiologic Studies Depression Scale (CES‐D)^20^ and a post void residual urine (PVR) was obtained. R‐FAS is scored from 0 to 27 with lower scores indicative of more favorable bowel function. All IIEF scores were obtained without pharmacologic or mechanical assistance. Potency was defined as an IIEF >12. CES‐D was integrated into our patient evaluation in September 2011.

Patients were evaluated by one of three age groups (≤59, 60‐69, and ≥70 years). A one‐way Anova was utilized to determine the differences across the three age groups for continuous variables and Chi‐square analysis was used to determine the differences of the categorical variables. OS and potency preservation was evaluated with a Kaplan‐Meier survival curve and competing risk analysis was used to determine TI. For all tests a *P* value ≤.05 was considered statistically significant. Statistical analysis was performed using STATA (Version 15.0, STATA Corp, LP, College Station, Texas).

## 3 RESULTS

The study population consisted of 305 consecutive, prospectively evaluated AS patients. All patients underwent TTMB as a staging procedure for a TRUS or transperineal diagnosed Gleason score 6 (3+3) adenocarcinoma of the prostate gland, a persistently elevated PSA and/or the presence of ASAP (Table 1). Two hundred and ninety patients (95.1%) were enrolled with Gleason score 3 + 3 histology while 15 patients (4.9%) had Gleason score 3 + 4. The overall mean and median follow‐up was 5.2 years and 5.5 years, respectively (range 1‐14 years). Of the patients, 298 (97.7%) were white and 7 (2.3%) were black. The mean pre‐diagnosis PSA was 5.86 ng/mL (range 0.50‐22.0 ng/mL). Sixteen patients were enrolled with a prediagnosis PSA of 10‐19.9 ng/mL and two patients presented with a PSA >20.0 ng/mL. The mean prostate volume was 61.1 cm^3^ (volumetric evaluation) with a median of 59 TTMB cores. When TRUS and TTMB biopsies cores were combined, a mean of 71 cores were obtained with a mean of three positive cores. Younger patients (≤59 years of age) presented with a lower pre‐diagnosis PSA, a smaller prostate volume, a higher incidence of Gleason score 6 histology, a lower positive core percentage and were less likely to present with hypertension, coronary artery disease, diabetes or hypercholestoremia.

**TABLE 1:** Clinical parameters of the study population, stratified by age

Continuous variables	≤59 n = 73		60‐69 n= 145		≥70 n=87		Total n=305		*P‐*value*
	Median	Mean	Median	Mean	Median	Mean	Median	Mean	
Pre‐TTMB PSA	4.5	4.82	5	5.78	6.1	6.90	5.1	5.86	**<.001**
Prostate volume									
Volumetric	45.5	50.0	53.3	60.3	62.3	69.9	56.5	61.1	**<.001**
Ellipsoid	36.9	43.2	45.5	51.1	55.8	62.0	46.6	52.4	**<.001**
Transition zone	13.5	30.1	28.5	29.0	29.4	33.6	20.2	25.9	**<.001**
PSA density	0.111	0.126	0.113	0.120	0.104	0.126	0.111	0.127	.994
BMI	29.8	29.7	28.5	29.0	28.5	28.9	28.7	29.2	.219
TRUS biopsy sessions	1	1.34	1	1.28	1	1.52	1	1.36	.112
TRUS biopsy cores	13	17.6	12	17.4	12	19.0	12	17.9	.772
TRUS Pos. cancerous cores	1	1.37	1	1.45	1	1.24	1	1.37	.698
TTMB number of cores	54	48.3	59	54.4	60	57.5	59	53.9	**.001**
Number of cores/Patient	66	62.8	71	68.0	71	73.2	69	68.3	**.004**
Number of pos. cores/Patient	2	3.56	2	3.47	3	4.18	2	3.70	.374
Malignant involvement (%)	4.5	4.86	5	6.17	5	8.19	5	6.47	**.008**
Follow‐up (years)	4.9	5.4	5.5	5.8	4.8	5.2	5.2	5.5	.329

Categorical variables	Count	(%)	Count	(%)	Count	(%)	Count	(%)	*P‐*value**
Gleason score									.063
3+3	72	98.6%	139	95.7%	79	90.8%	290	95.1%	
3+4	1	1.4%	6	4.3%	8	9.2%	15	4.9%	
TTMB cancer symmetry									.624
–	14	19.2%	31	21.4%	12	13.8%	57	18.7%	
Unilateral	40	54.8%	71	48.9%	49	56.3%	160	52.5%	
Bilateral	19	26.0%	43	29.7%	26	29.9%	88	28.9%	
Perineural invasion	5	6.9%	9	6.2%	6	6.9%	20	6.6%	.973
Hypertension	39	53.4%	93	64.1%	69	73.3%	201	65.9%	**.002**
Coronary artery disease	6	8.8%	22	16.3%	19	22.9%	47	16.4%	.068
Diabetes	5	6.9%	31	21.4%	22	25.3	58	19.0%	**.008**
Hypercholesterolemia	24	34.8%	75	55.2%	50	60.2	149	51.7%	**.004**
Tobacco									.633
Never	38	52.1%	66	45.5%	30	34.5%	134	43.9%	
Former	22	30.1%	58	40.0%	53	60.9%	133	43.6%	
Current	13	17.8%	21	14.5%	4	4.6%	38	12.5%	
Testosterone									.133
Lower 1/3	59	80.8%	102	70.3%	66	75.9%	227	74.4%	
Middle 1/3	11	15.1%	16	11.0%	7	8.0%	34	11.2%	
Upper 1/3	3	4.1%	27	18.7%	14	16.1%	44	14.4%	
TURP	2	2.7%	6	4.1%	4	4.6%	12	3.9%	.822
Race									.446
White	70	95.9%	142	97.9%	86	98.9%	298	97.7%	
Black	3	4.1%	3	97.9%	1	1.1%	7	2.3%	

* Two‐way ANOVA;

** Chi‐Square.

**FIGURE 1:**
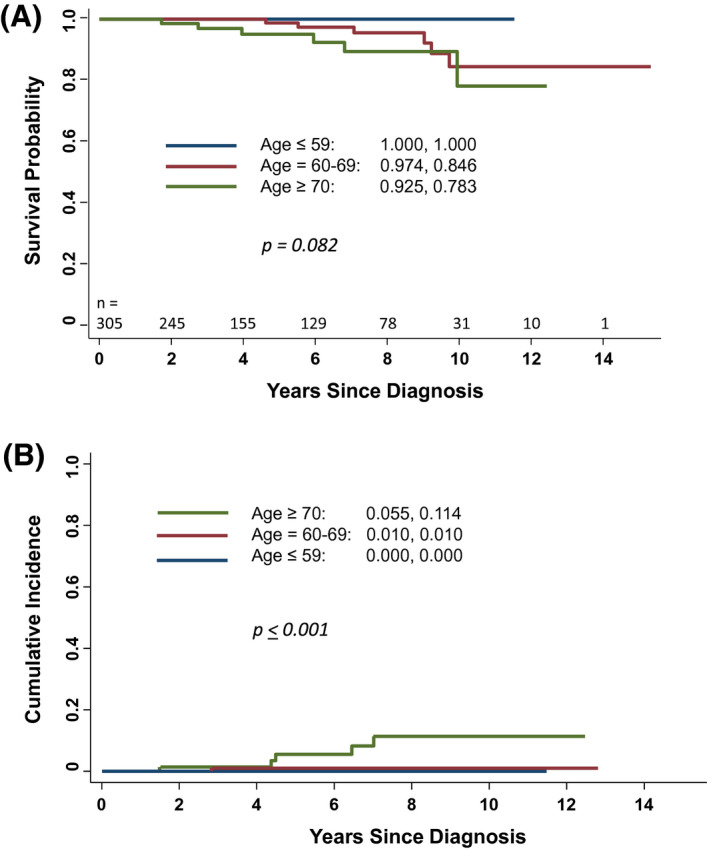
A, Overall survival at 6 years, stratified by age, B, Therapeutic intervention at 6 and 10 years, stratified by age

OS at 10 years was 100%, 84.6%, and 78.3% in patients ≤59, 60‐69, and ≥70 of age (*P* = .082) (Figure 1A). Therapeutic intervention at 10 years was 0%, 1%, and 11.4% for patients ≤59, 60‐69, and ≥70 years of age (*P *< .001) (Figure 1B). To date, no patient has developed distant metastases. Of the patients who have failed, the mean and median time to therapeutic intervention was 5.03 years and 4.71 years, respectively. Failed patients presented with larger prostate and transition zone volumes and were older in age. Of the patients who have not required treatment, 98.0% (291 patients) have a PSA doubling time of more than 3 years. Twelve patients have died with none dying of prostate cancer. Deaths have been a result of coronary artery disease (four patients), second malignancies (six patients), Parkinson’s disease (one patient), and trauma (one patient). In terms of TTMB‐related morbidity, no patient required hospitalization, developed sepsis, or required a transurethral resection of the prostate gland. Two hundred and fifty four patients (83.8%) did not require post‐TTMB catheterization, while 28 patients (9.2%) required overnight catheterization alone and 23 patients (7.5%) required a urinary catheter for 2‐4 days. No patient required a catheter beyond 4 days.

**FIGURE 2:**
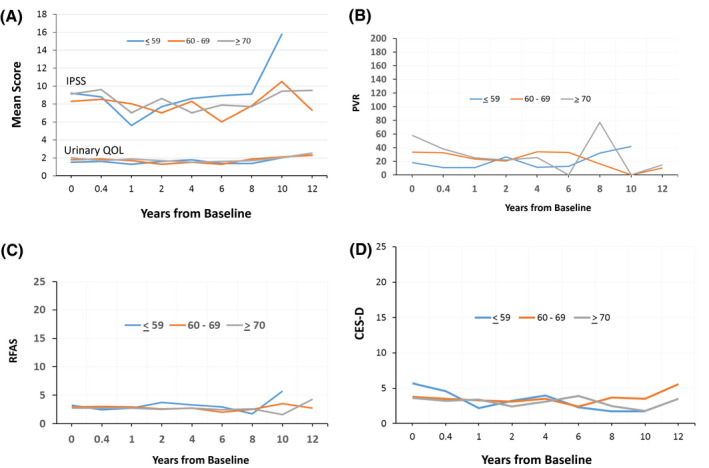
A, Mean (IPSS) and Urinary QOL stratified by age, B, Mean PVR stratified by age, C, RFAS stratified by age, D, CES‐D stratified by age

**FIGURE 3:**
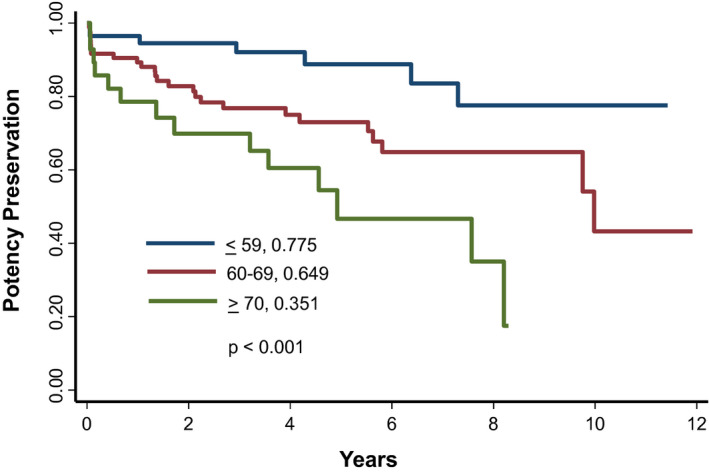
Potency preservation stratified by age

In terms of quality of life, no significant change in IPSS, urinary QOL, PVR, rectal function, or depression scores were noted throughout the duration of the study (Figure 2). At 8 years, 77.5%, 64.9%, and 35.1% of patients ≤59, 60‐69, and ≥70 years of age remained potent (*P* < .001) (IIEF>12 without pharmacologic or mechanical assistance) (Figure 3).

## 4 DISCUSSION

Successful AS outcomes are dependent on patient selection. However, the possible misdiagnosis of aggressive cancers could result in late prostate cancer mortality, especially in young patients. Approximately 1/3 of AS men proceed to therapeutic intervention within the first few years of follow‐up most likely as a result of sampling error of TRUS biopsy. For this reason, we have utilized TTMB as a staging procedure for AS patient selection because of its ability to accurately identify high‐grade prostate cancers.^10^ The majority of clinically significant prostate cancers missed at TRUS biopsy are located in the anterior gland which is a region easily accessible by TTMB.^13^, ^14^

AS is an appealing strategy in younger patients because these patients have better baseline urinary and sexual function compared to older patients. Until recently, younger patients with favorable prostate cancer were most likely treated with definitive local therapy. However, recent trends demonstrate an increased acceptance of AS management in these younger patients.^8^ It has been reported that younger men managed with AS are not at increased risk for progression.^7^, ^21^ Druskin and colleagues reported a 5 year incidence of biopsy grade reclassification to grade group 3 or greater of 4%, 7%, and 14% in men younger than 60, 60‐69, and 70 years of age or older (*P* < .001).^21^ This is consistent with the incidence of therapeutic intervention in our series. Figure 1B illustrates therapeutic intervention rates of 0%, 1.0% and 11.4% of men ≤ 59, 60‐69, and ≥ 70 of age at 10 years (*P* ≤ .001).

Recent AS studies demonstrate that PSA kinetics predict follow‐up biopsy outcomes in selected situations.^22^, ^23^, ^24^ Iremashvili et al studied 314 AS patients who had undergone at least 1 repeat TRUS biopsy.^22^ Beginning with the fourth TRUS biopsy, PSA kinetics (PSAV and PSADT) were associated with risk of progression and were independent of baseline characteristics. In their series, each TRUS biopsy consisted of 10‐12 cores with a resultant 40‐50 cores for all 4‐5 biopsy sessions which is less than the mean of 71 biopsy cores at the time of enrollment in our series. In addition, a low risk of progression to a lethal phenotype is extremely important since we do not perform routine follow‐up biopsies. Tosoian et al reported that in favorable risk prostate cancer, the risk of progression to a lethal phenotype is low (0.4%) in the decade following diagnosis.^9^ Importantly, a review of Gleason score 6 radical prostatectomy (RP) specimens confirmed the absence of metastatic potential in these lesions.^25^ Following RP with Gleason score 6 histology a 0.2% cancer death rate was reported.

A possible shortcoming of our AS protocol is the absence of routine follow‐up prostate biopsies. In our series, repeat TTMB biopsies are reserved for patients with an abnormal DRE, a PSA doubling time <3 years and/or patient preference. Since our TTMB selected patients have undergone extensive sampling of all regions of the prostate including the anterior gland, it is conceivable that additional biopsies are unnecessary for the majority of patients, especially in light of the fact that TTMB results closely mimic RP pathology.^10^ Although biopsy grade reclassification remains the most common trigger for curative intervention among AS patients, a sizeable proportion of patients never undergo a follow‐up biopsy secondary to patient preference despite recommendations for biopsies every 1 to 4 years.^1^, ^3^, ^9^ Only 30% of men on AS in the PRIAS (Prostate Cancer Research International Active Surveillance) series followed for more than 4 years had undergone all of the biopsies recommended by study protocol.^26^ In the future, genomic testing and MRI will play greater roles in patient selection and follow‐up.

Strengths of our study include its prospective nature, all patients underwent the same intensive initial biopsy procedure prior to AS enrollment and underwent consistent and complete follow‐up. Weaknesses of our study is that prostate cancer has a long natural history and additional follow‐up of this cohort will be mandatory to determine durability. The necessity of general anesthesia and operating room time has limited the widespread adoption of TTMB. In addition, consistent with the John’s Hopkins study our series is enriched with white patients (97.7%). As such, our results may not be applicable to minority patients.^4^

## 5 CONCLUSIONS

Within the confines of the follow‐up of our series, younger patients were less likely to proceed to therapeutic intervention. In addition, patient age did not adversely impact QOL outcomes.

## CONFLICT OF INTEREST

None.
